# Association between serum uric acid and prostate cancer mortality in androgen deprivation therapy: A population‐based cohort study

**DOI:** 10.1002/cam4.6344

**Published:** 2023-07-16

**Authors:** Yan Hiu Athena Lee, Jeffrey Shi Kai Chan, Chi Ho Leung, Jeremy Man Ho Hui, Edward Christopher Dee, Kenrick Ng, Kang Liu, Tong Liu, Gary Tse, Chi Fai Ng

**Affiliations:** ^1^ Cardio‐oncology Research Unit Cardiovascular Analytics Group, PowerHealth Limited Hong Kong China; ^2^ SH Ho Urology Centre, Department of Surgery, Faculty of Medicine The Chinese University of Hong Kong Hong Kong China; ^3^ Department of Radiation Oncology Memorial Sloan Kettering Cancer Center New York New York USA; ^4^ Department of Medical Oncology University College London Hospitals NHS Foundation Trust London UK; ^5^ Tianjin Key Laboratory of Ionic‐Molecular Function of Cardiovascular Disease, Department of Cardiology, Tianjin Institute of Cardiology Second Hospital of Tianjin Medical University Tianjin China; ^6^ Kent and Medway Medical School Canterbury Kent UK; ^7^ School of Nursing and Health Studies Hong Kong Metropolitan University Hong Kong China

**Keywords:** clinical cancer research, epidemiology, hormone therapy, prognostic factor, prostate cancer

## Abstract

**Objective:**

This population‐based study examined the association between baseline uric acid (UA) and prostate cancer (PCa)‐related mortality amongst PCa patients receiving androgen deprivation therapy (ADT).

**Methods:**

Adults with PCa who received ADT in Hong Kong between December 1999 and March 2021 were identified. Patients with missing baseline UA were excluded. Patients were followed up until September 2021. The outcome was PCa‐related mortality.

**Results:**

Altogether, 4126 patients (median follow‐up 3.1[interquartile range 1.4–6.0] years) were included. A J‐shaped association was observed between baseline UA level and PCa‐related mortality risk, with a direct association in those with mean(0.401 mmol/L) or above‐mean baseline UA levels (hazard ratio (HR) per standard deviation‐increase 1.35 [95% confidence interval 1.21,1.51], *p* < 0.001), and an inverse association in those with below‐mean baseline UA levels (HR 0.78[0.67,0.92], *p* = 0.003). The former remained significant on competing risk regression, but not the latter.

**Conclusions:**

A J‐shaped relationship between baseline UA level and PCa‐related mortality risk was identified. This study was mainly limited by potential unmeasured and residual confounders. Further validation studies are warranted.

## Graphical abstract

Amongst patients with prostate cancer undergoing androgen deprivation therapy, a J‐shape relationship exists between baseline uric acid level and the risk of prostate cancer‐related mortality, with both high and low uric acid levels being associated with similarly elevated risks when compared against normal uric acid levels.

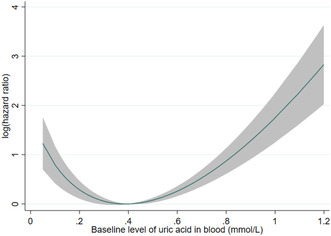

## 1 INTRODUCTION

Gout and hyperuricaemia have been associated with higher cancer incidence and mortality,^1^, ^2^ with particularly strong associations between gout and incident prostate cancer (PCa).^1^ Laboratory evidence has demonstrated that higher uric acid (UA) levels promote PCa cell growth.^3^ It is thus plausible that UA levels are linked to aggressiveness and progression of PCa and thereby influence the clinical risk of PCa‐related mortality. However, clinical evidence is lacking in this regard. As UA level testing is readily accessible and inexpensive, it may be a viable prognostic tool for patients with PCa if UA levels are indeed correlated with PCa‐related mortality. Therefore, this population‐based study examined the association between baseline UA and PCa‐related mortality amongst PCa patients receiving androgen deprivation therapy (ADT). These patients were selected for this exploratory study as ADT is only indicated in advanced diseases^4^ where PCa‐related mortality is relatively common, increasing statistical power.

## 2 METHODS

This prospective cohort study was approved by The Joint Chinese University of Hong Kong‐New Territories East Cluster Clinical Research Ethics Committee and conducted in accordance with the Declaration of Helsinki. Patient consent was waived as deidentified data were used. All underlying data are available upon reasonable request to the corresponding authors.

### 2.1 Source of data

Data were obtained from the Clinical Data Analysis and Reporting System, a prospective population‐based database of patients attending public healthcare institutions in Hong Kong, China. Diagnoses were recorded by *International Classification of Diseases, Ninth revision* (ICD‐9) codes (Table S1), as ICD‐10 codes have not been implemented in CDARS to date. Mortality data was obtained from the linked Hong Kong Death Registry, a governmental registry of the death records of all Hong Kong citizens; causes of death were recorded using ICD‐9 or ICD‐10 codes. Both databases have been used in previous studies.^5^, ^6^

### 2.2 Patient population

Patients aged ≥18 years old with PCa who received any ADT (gonadotropin‐releasing hormone agonists or antagonists, or bilateral orchidectomy) during 1/12/1993–31/3/2021 were included. Patients with missing baseline UA level were excluded. The endpoint was PCa‐related mortality. Patients were followed up from the date of ADT initiation until death or 31/9/2021, whichever earlier. PCa diagnosis was ascertained using ICD‐9 codes (Table S1), and PCa‐related mortality was identified using ICD‐9 and ICD‐10 codes (Table S2).

### 2.3 Variables collected

The following baseline characteristics were collected for all patients: age, the type of ADT, serum uric acid level, comorbid conditions (stroke, hypertension, diabetes mellitus, ischaemic heart disease, chronic kidney disease, atrial fibrillation, dyslipidaemia, and gout), prior radiotherapy, prior radical prostatectomy, medication prescriptions (angiotensin‐converting enzyme inhibitors or angiotensin receptor blockers, metformin, sulfonylurea, dipeptidyl peptidase‐4 inhibitors, glucagon‐like peptide 1 receptor agonists, insulins, beta‐blockers, dihydropyridine calcium channel blockers, statins, antiplatelets, anticoagulants, and UA‐lowering medications). Furthermore, ever‐prescription of androgen receptor signaling inhibitors and/or chemotherapy was recorded as a surrogate for metastatic disease^4^; this was done to mitigate our database's limitation that cancer staging, histology, and risk profile were unavailable. These variables were used for multivariable adjustments. The duration of ADT amongst those who only received gonadotropin‐releasing hormone agonist or antagonist was also recorded but not adjusted for to avoid immortal time bias.

### 2.4 Statistical analyses

**FIGURE 1:**
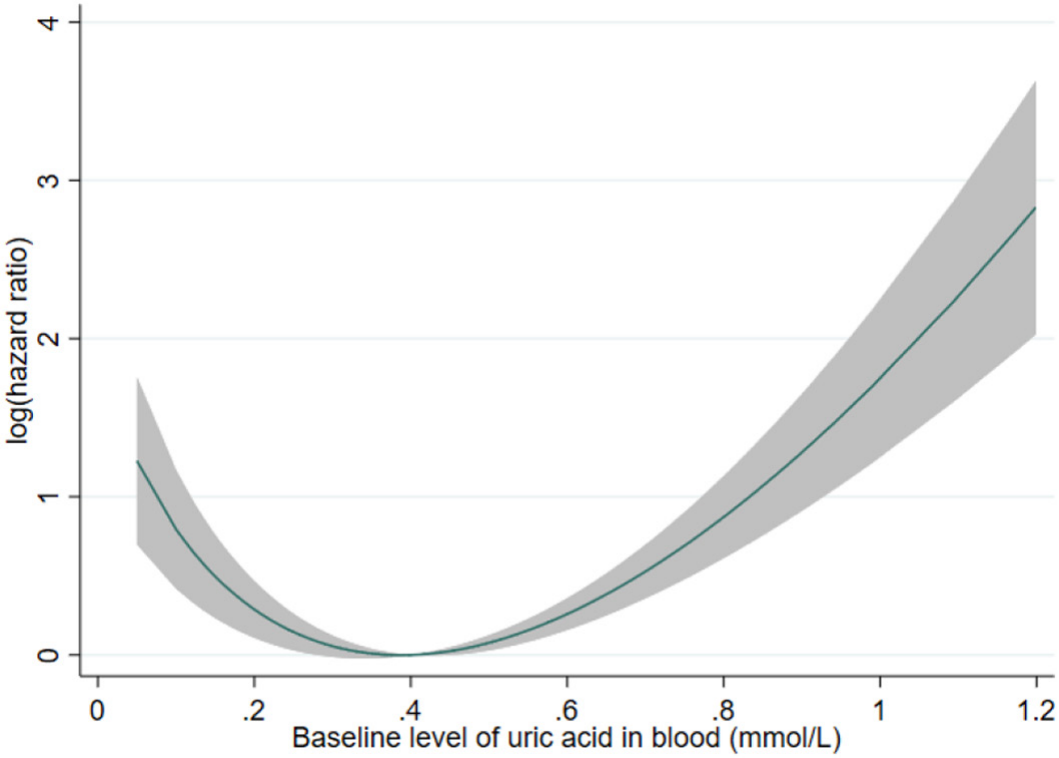
Fractional polynomial curve showing the association between baseline uric acid level and the risk of prostate cancer‐related mortality across the observed range of baseline uric acid level.

Continuous variables were expressed as median and interquartile range. Due to the nature of the database, and as patients with missing baseline UA levels were excluded, there were no missing values in this study. Multivariable Cox regression adjusting for covariates (as abovementioned) was used to evaluate the relationship between baseline UA level and the endpoint, with hazard ratio (HR) and 95% confidence intervals (CIs) as summary statistics. A fractional polynomial curve was used to explore such relationship across the observed range of baseline uric acid levels. As a J‐shaped relationship was observed (Figure 1), the patients were divided into two subgroups: one with baseline UA level above the mean (0.401 mmol/L), and another with baseline UA level below or equal to the mean. Multivariable Cox regression was then carried out in each subgroup separately. The baseline UA level was analyzed as a standardized variable, such that the HR represents the change in risk per standard deviation increase in baseline UA level.

For each of these groups, four a priori subgroup analyses were performed. First, to assess the effects across patients of different ages, patients were grouped into those aged 75 years old or above and those younger than 75 years old. Second, as gout is intimately related to UA levels and may be a confounder, patients were grouped by any prior diagnosis of gout. Similarly, UA‐lowering medications affect UA levels and thus may be a confounder, and a subgroup analysis was done accordingly, with grouping by the baseline use of UA‐lowering medication(s). Finally, to explore the respective effects of UA levels in metastatic and non‐metastatic PCa, a subgroup analysis was performed for metastatic vs non‐metastatic PCa, with ever‐prescription of androgen receptor signaling inhibitor(s) or chemotherapy used as a surrogate and thus grouping criterion for metastatic disease.^4^

As the high rate of mortality may bias hazards estimated by conventional survival analyses, an a priori sensitivity analysis was performed using competing risk analysis with the Fine‐Gray subdistribution model. Non‐PCa‐related mortality was the competing event. Sub‐hazard ratios with 95% CIs were used as summary statistics.

To facilitate clinical translation of our findings, a post hoc analysis was performed where uric acid levels were referenced against the male normal range of 0.24–0.51 mmol/L and categorized into low‐ (<0.24 mmol/L), normal‐ (0.24–0.51 mmol/L), and high‐ (>0.51 mmol/L) uric acid categories. All patients were then analyzed with uric acid as these categories, without the above separation by the mean uric acid level, using multivariable Cox regression.

All *p*‐values were two‐sided, with *p* < 0.05 considered statistically significant. All analyses were performed on Stata (v16.1, StataCorp LLC).

## 3 RESULTS

Altogether, 13,537 patients with PCa receiving ADT were identified. After excluding 9411 patients without baseline UA level, 4126 patients were analyzed (Figure S1; median age 76.6 [interquartile range 70.8–82.1] years old), whose baseline characteristics are summarized in Table S3. Over a median follow‐up of 3.1[1.4–6.0] years, 2691 (65.2%) died, with 1036 (25.1%) having PCa‐related mortality and 1655 (40.1%) having non‐PCa‐related mortality.

A J‐shaped relationship was observed between UA levels and the risk of PCa‐related mortality (Figure 1). In those with mean/above‐mean UA levels (≥0.401 mmol/L; *N* = 1852), higher UA levels were associated with higher risk of PCa‐related mortality (hazard ratio (HR) per SD‐increase 1.35 [95% CI 1.21,1.51], *p* < 0.001). Subgroup analyses (Table S4) found directionally consistent associations in all subgroups, with vastly overlapping CIs suggesting that none of the stratified variables had any meaningful effect on the association between above‐mean UA levels and the risk of PCa‐related mortality. Sensitivity analysis using competing risk regression consistently showed a direct association between baseline UA level and PCa‐related mortality (sub‐hazard ratio (SHR) 1.26[1.11,1.42], *p* < 0.001).

In those with below‐mean UA levels (<0.401 mmol/L; *N* = 2274), lower UA levels were associated with higher risk of PCa‐related mortality (HR 0.78[0.67,0.92], *p* = 0.003). Subgroup analyses (Table S4) found directionally consistent associations in all subgroups, with vastly overlapping CIs suggesting that none of the stratified variables had any meaningful effect on the association between below‐mean UA levels and the risk of PCa‐related mortality. The association between UA level and PCa‐related mortality approached significance on sensitivity analysis using competing risk regression (SHR 0.86[0.73,1.01], *p* = 0.071).

**FIGURE 2:**
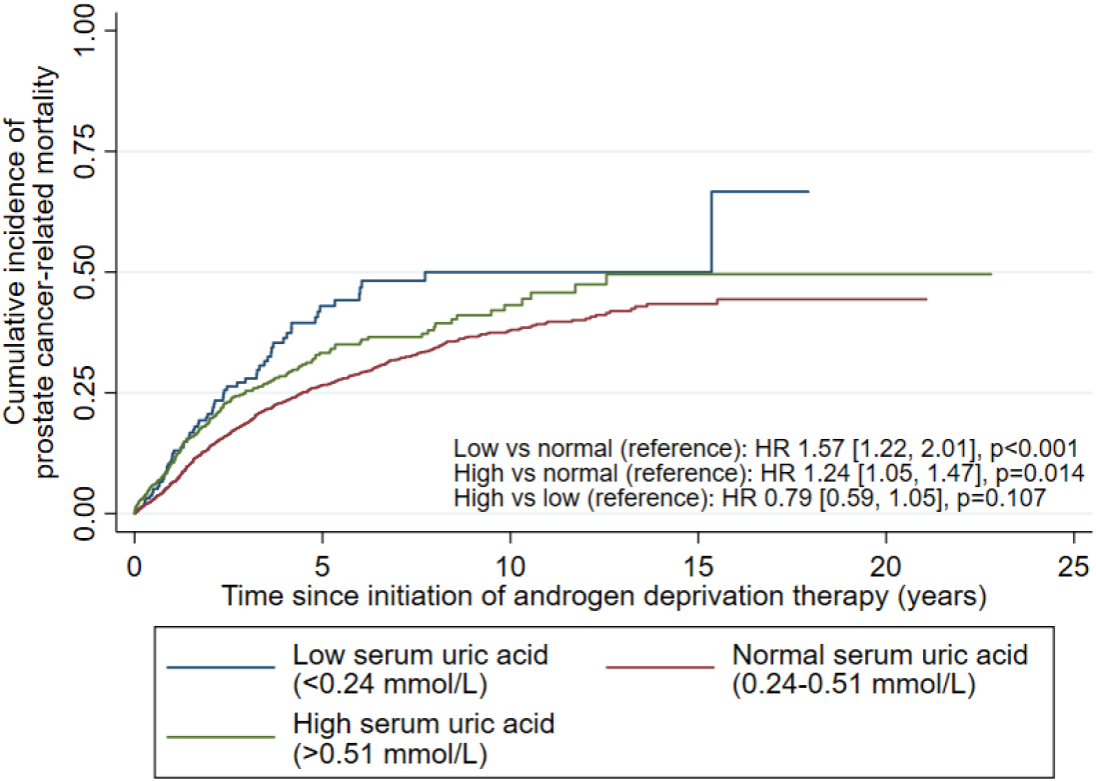
Kaplan–Meier cumulative incidence curve showing the cumulative incidence of prostate cancer‐related mortality amongst patients with low (<0.24 mmol/L), normal (0.24–0.51 mmol/L), and high (>0.51 mmol/L) baseline uric acid levels. Hazard ratios (HRs) shown were adjusted for covariates.

Compared to the normal‐UA group, both high‐UA (HR 1.24[1.05,1.47], *p* = 0.014) and low‐UA (HR 1.57[1.22,2.01], *p* < 0.001) groups had higher cumulative incidence of PCa‐related mortality (Figure 2), but not between the high‐ and low‐UA groups (HR 0.79[0.59,1.05], *p* = 0.107; referenced against low‐UA group). Both the high‐UA (SHR 1.20[1.01,1.44], *p* = 0.039) and low‐UA groups had significantly higher cumulative incidence of PCa‐related mortality on competing risk regression (SHR 1.36[1.06,1.75], *p* = 0.015); the high‐ and low‐UA groups had similar risks on competing risk regression (SHR 0.88[0.66,1.18], *p* = 0.400).

## 4 DISCUSSION

As far as we know, this was one of the first studies exploring the prognostic value of baseline UA levels in patients with PCa. Mechanistically, in those with mean/above‐mean baseline UA levels, the direct association between baseline UA level and PCa‐related mortality UA levels may be explained by PCa progression due to UA‐induced activin insensitivity, suppressed UA transporter expression, and UA‐related intraprostatic inflammation.^3^, ^7^, ^8^ Meanwhile, in those with below‐mean baseline UA levels, the inverse association may be explained by the low UA levels reflecting frailty. Hypouricaemia was associated with frailty,^9^ which was associated with higher risks of both all‐cause and cancer‐specific mortality in patients with PCa.^10^

Clinically, our findings suggest that baseline UA level may be used for prognosticating patients with PCa receiving ADT, warranting similar investigations in broader cohorts of patients with PCa. Additionally, although probenecid may abolish UA's effects on PCa cells in vitro,^3^ UA‐lowering medications did not meaningfully modify the captioned associations in our study. Further studies are needed to investigate more granularly the effects of UA‐lowering medications on PCa‐related outcomes, and whether UA level is a meaningful treatment target in PCa.

### 4.1 Strengths and limitations

Using prospective, population‐based data, our findings were representative and likely generalizable to other Asian/Chinese cohorts of patients with PCa receiving ADT. Nonetheless, this study's observational nature predisposes to unmeasured and residual confounding. Additionally, many were excluded for missing baseline UA level, likely selecting for patients with indications for UA testing and limiting generalizability. Moreover, staging data were not available, which we partially mitigate by using ever‐prescription of ARSI/chemotherapy as a surrogate for metastatic disease.^4^ Lastly, individual data adjudication was impossible. Nonetheless, the databases have been demonstrated to have good data completeness and accuracy.^6^

## 5 CONCLUSIONS

A J‐shaped relationship between baseline UA level and PCa‐related mortality risk was identified. Further validation studies are warranted.

## AUTHOR CONTRIBUTIONS

**Yan Hiu Athena Lee:** Conceptualization (equal); data curation (equal); methodology (equal); writing – original draft (equal); writing – review and editing (equal). **Jeffrey Shi Kai Chan:** Conceptualization (equal); formal analysis (lead); methodology (lead); visualization (lead); writing – original draft (equal); writing – review and editing (equal). **Chi Ho Leung:** Methodology (supporting); writing – review and editing (supporting). **Jeremy Man Ho Hui:** Investigation (equal); writing – review and editing (supporting). **Edward Christopher Dee:** Writing – review and editing (supporting). **Kenrick Ng:** Writing – review and editing (supporting). **Kang Liu:** Data curation (equal); resources (equal); writing – review and editing (supporting). **Tong Liu:** Funding acquisition (supporting); supervision (equal); writing – review and editing (supporting). **Gary Tse:** Funding acquisition (lead); methodology (supporting); resources (equal); supervision (equal); writing – review and editing (supporting). **Chi‐Fai Ng:** Conceptualization (equal); funding acquisition (supporting); supervision (equal); writing – review and editing (equal).

## FUNDING INFORMATION

This work was partly supported by the Research Matching Grant (reference number 8601454), the Tianjin Key Medical Discipline (Specialty) Construction Project (Project number: TJYXZDXK‐029A), and a grant from Hong Kong Metropolitan University (Project Reference No. RIF/2022/2.2). The funders played no role in any part of this study.

## CONFLICT OF INTEREST STATEMENT

ECD is funded in part through the Cancer Center Support Grant from the National Cancer Institute (P30 CA008748). All other authors have no conflict of interest to report.

## ETHICS STATEMENT

This prospective cohort study was approved by The Joint Chinese University of Hong Kong‐New Territories East Cluster Clinical Research Ethics Committee and conducted in accordance with the Declaration of Helsinki. Patient consent was waived as deidentified data were used.

## Supporting information


Table S1.

## Data Availability

All underlying data are available upon reasonable request to the corresponding authors.
