# Supplementary material for: Association between serum uric acid and prostate cancer mortality in androgen deprivation therapy: A population‐based cohort study
Source: Cancer Med. 2023 Jul 16;12(16):17056–60. doi: 10.1002/cam4.6344 (PMC10501275; doi:10.1002/cam4.6344)

**Supplementary material**

**Supplementary Table 1.** International Classification of Diseases, Ninth Revision (ICD-9) codes used to identify outcomes and co-morbidities. All hereby listed codes include the corresponding sub-codes.

| **Condition** | **ICD-9 codes** |
| --- | --- |
| Prostate cancer | 185 |
| Heart failure | 428 |
| Myocardial infarction | 410 |
| Diabetes mellitus | 250 |
| Hypertension | 401 402 403 404 405 437.2 |
| Atrial fibrillation | 427.31 |
| Stroke | 430 431 432 433 434 435 |
| Ischaemic heart disease | 410 411 412 413 414 |
| Chronic kidney disease | 582 585 586 |
| Chronic liver disease | 456.0 456.1 456.20 456.21 571 572.2 572.3 572.4 572.5 572.6 572.7 572.8 |
| Dyslipidaemia | 272.0 272.1 272.2 272.3 272.4 |

**Supplementary Table 2.** International Classification of Diseases, Ninth Revision (ICD-9) and Tenth Revision (ICD-10) codes used to identify the cause of death. All hereby listed codes include the corresponding sub-codes.

| **Type of mortality** | **ICD codes** |
| --- | --- |
| Prostate cancer-related mortality | ICD-9: 185  ICD-10: C61 |

**Supplementary Table 3.** Characteristics of included patients. Median and interquartile ranges are displayed for continuous variables. All variables refer to the patients’ baseline status, except the duration of use of gonadotropin-releasing hormone agonist / antagonist, and ever- prescription of ARSI and/or chemotherapy.

| Age, years | 76.6 [70.8-82.1] |
| --- | --- |
| GnRH agonist or antagonist only, N (%) | 2303 (55.8) |
| Duration of use, years | 1.67 [0.50-3.14] |
| Bilateral orchiectomy only, N (%) | 1425 (34.5) |
| GnRH agonist or antagonist followed by bilateral orchiectomy, N (%) | 398 (9.7) |
| Hypertension, N (%) | 1515 (36.7) |
| Diabetes mellitus, N (%) | 1154 (28.0) |
| Ischaemic heart disease, N (%) | 599 (14.5) |
| Stroke, N (%) | 483 (11.7) |
| Chronic kidney disease, N (%) | 253 (6.1) |
| Atrial fibrillation, N (%) | 278 (6.7) |
| Dyslipidaemia, N (%) | 563 (13.7) |
| Gout, N (%) | 927 (22.5) |
| Radiotherapy, N (%) | 138 (3.3) |
| Radical prostatectomy, N (%) | 1135 (27.5) |
| ACEI/ARB, N (%) | 1397 (33.9) |
| Metformin, N (%) | 530 (12.9) |
| Sulfonylurea, N (%) | 735 (17.8) |
| DPP4 inhibitor, N (%) | 76 (1.8) |
| GLP1RA, N (%) | 1 (0.0) |
| Insulin, N (%) | 359 (8.7) |
| Beta-blocker, N (%) | 1749 (42.4) |
| Dihydropyridine CCB, N (%) | 2177 (52.8) |
| Statin, N (%) | 1451 (35.2) |
| Antiplatelet, N (%) | 1254 (30.4) |
| Anticoagulant, N (%) | 207 (5.0) |
| Uric acid-lowering medication, N (%) | 610 (14.8) |
| Ever-prescription of ARSI and/or chemotherapy, N (%) | 1647 (39.9) |
| Baseline uric acid level, mmol/L | 0.390 [0.328-0.460] |

ACEI, angiotensin-converting enzyme inhibitor. ARB, angiotensin receptor blocker. ARSI, androgen receptor signalling inhibitor. CCB, calcium channel blocker. DPP4, dipeptidyl peptidase-4. GLP1RA, glucagon-like peptide 1 receptor agonists.

**Supplementary Table 4.** Results of subgroup analyses in patients with baseline uric acid level at or above mean (0.401 mmol/L).

|  | Number of patients | Hazard ratio [95% confidence interval] |
| --- | --- | --- |
| Age ≥75 years old | 1124 | 1.40 [1.22, 1.61] |
| Age <75 years old | 728 | 1.25 [1.05, 1.50] |
| With metastatic prostate cancer | 670 | 1.31 [1.10, 1.57] |
| Without metastatic prostate cancer | 1182 | 1.38 [1.20, 1.59] |
| With prior diagnosis of gout | 501 | 1.18 [0.94, 1.48] |
| No prior diagnosis of gout | 1351 | 1.41 [1.24, 1.60] |
| With prior prescription of urate-lowering medication(s) | 286 | 1.22 [0.91, 1.61] |
| No prior prescription of urate-lowering medication(s) | 1566 | 1.39 [1.24, 1.57] |

**Supplementary Table 5.** Results of subgroup analyses in patients with baseline uric acid level below mean (0.401 mmol/L).

|  | Number of patients | Hazard ratio [95% confidence interval] |
| --- | --- | --- |
| Age ≥75 years old | 1273 | 0.78 [0.62, 0.97] |
| Age <75 years old | 1001 | 0.79 [0.62, 1.01] |
| With metastatic prostate cancer | 977 | 0.75 [0.59, 0.95] |
| Without metastatic prostate cancer | 1297 | 0.83 [0.66, 1.03] |
| With prior diagnosis of gout | 426 | 0.81 [0.53, 1.23] |
| No prior diagnosis of gout | 1848 | 0.79 [0.66, 0.94] |
| With prior prescription of urate-lowering medication(s) | 324 | 0.75 [0.47, 1.19] |
| No prior prescription of urate-lowering medication(s) | 1950 | 0.79 [0.67, 0.94] |

**Supplementary Figure 1.** Study flow diagram. GnRH, gonadotropin-releasing hormone.


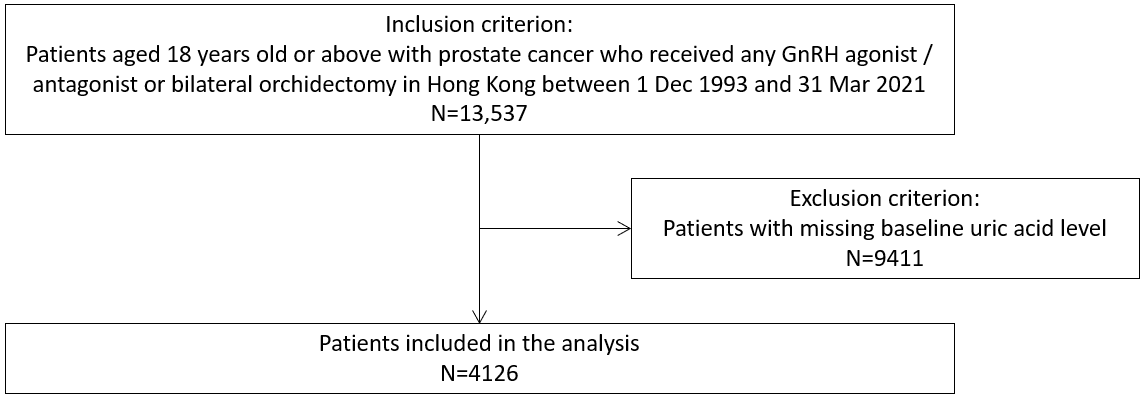

Supplement: Supplementary file 1 — Table S1. [file CAM4-12-17056-s001.docx]
